# Supplementary material for: Pancreatic Cancer Cells Induce MicroRNA Deregulation in Platelets
Source: Int J Mol Sci. 2022 Sep 28;23(19):11438. doi: 10.3390/ijms231911438 (PMC9569638; doi:10.3390/ijms231911438)
Supplement: Supplementary file 1 [file ijms-23-11438-s001.zip › ijms-1924363-SM.pdf]

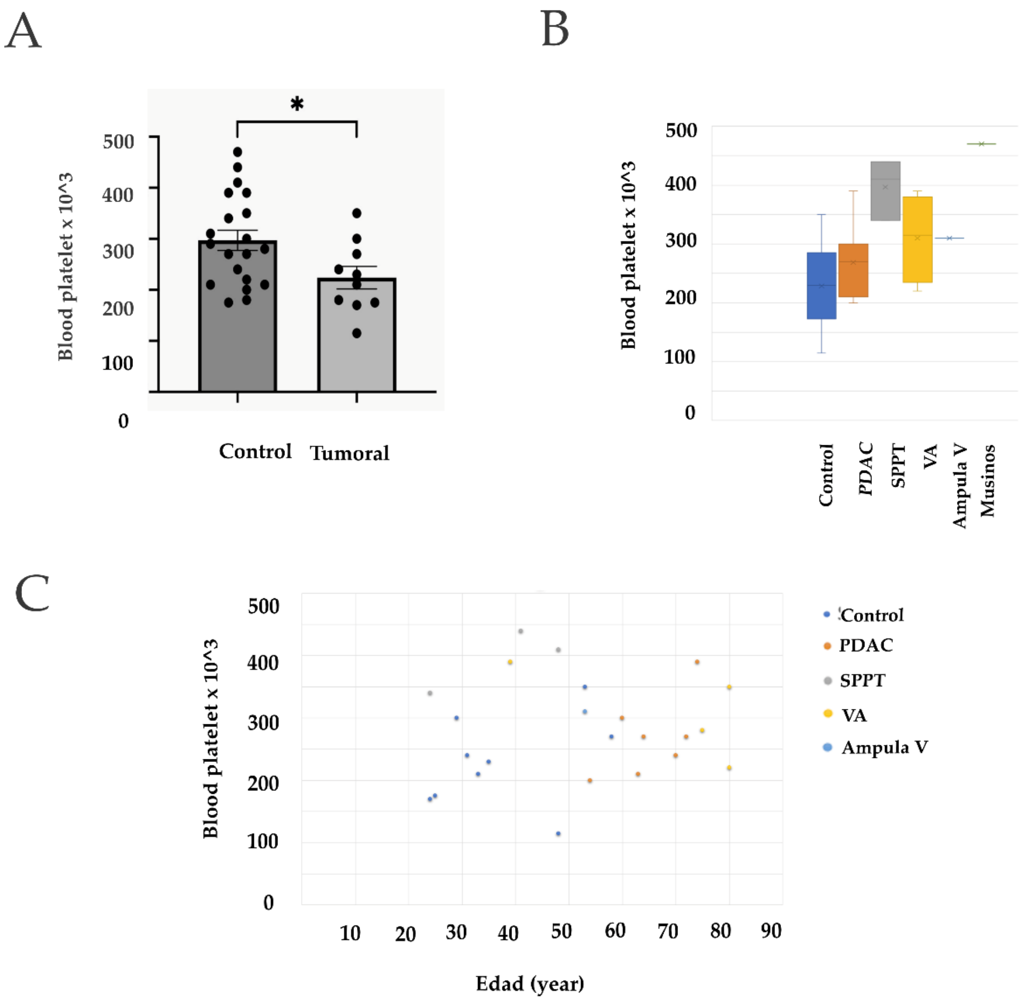

Supplementary Figure S1 1 Number of platelets in healthy people and patients with pancreatic cancer (A). Number of platelets graphed by diagnosis (B). Platelet count in healthy people with pancreatic cancer according to their diagnosis (C)

Table S1 General data from patients from whom blood samples were collected to obtain platelets.

Sample	Sex	Age	Phatology report	Blood sample	Pancreatic drainaje sample	Blood platelet x 10^3/ul
1	Female	53	Mixed adenoneuroendocrine carcinome ampulla of vater	yes	yes	310
2	Female	24	Mucinous cystadenocarcinomas	yes	no	470
3	Male	74	PDAC	yes	yes	390
4	Male	60	PDAC	yes	yes	300
5	Female	72	PDAC	yes	no	270
6	Female	64	PDAC	yes	no	270
7	Female	54	PDAC	yes	no	200
8	Male	63	PDAC	yes	yes	210
9	Male	70	PDAC	yes	yes	240
10	Male	68	PDAC	yes	yes	ND
11	Male	70	PDAC	yes	yes	ND
12	Female	70	PDAC	yes	yes	ND
13	Female	48	Solid pseudo papillary tumor	yes	yes	410
14	Male	41	Solid pseudo papillary tumor	yes	no	440
15	Female	24	Solid pseudo papillary tumor	yes	no	340
16	Female	80	Villous adenoma	yes	no	350
17	Male	75	Villous adenoma	yes	no	280
18	Male	39	Villous adenoma	yes	no	390
19	Female	80	Villous adenoma	yes	yes	220
20	Female	64	Villous adenoma	yes	no	ND
21	Female	24	Control	yes	no	180
22	Female	24	Control	yes	no	170
23	Female	25	Control	yes	no	175
24	Female	29	Control	yes	no	300
25	Male	31	Control	yes	no	240
26	Male	33	Control	yes	no	210
27	Male	35	Control	yes	no	230
28	Female	48	Control	yes	no	115
29	Male	53	Control	yes	no	350
3	Female	58	Control	yes	no	270
